# Editorial: Foot-and-mouth disease epidemiology, vaccines and vaccination: moving forward

**DOI:** 10.3389/fvets.2023.1231005

**Published:** 2023-06-20

**Authors:** Alejandra Victoria Capozzo, Wilna Vosloo, Teresa de los Santos, Andrés M. Pérez, Mariano Pérez-Filgueira

**Affiliations:** ^1^Institute of Virology and Technical Innovations, CONICET-INTA, Buenos Aires, Argentina; ^2^Australian Centre for Disease Preparedness, Transboundary Disease Mitigation, Commonwealth Scientific and Industrial Research Organisation (CSIRO) Health and Biosecurity, Geelong, VIC, Australia; ^3^Plum Island Animal Disease Center, Agricultural Research Service, U.S. Department of Agriculture, Greenport, NY, United States; ^4^Department of Veterinary Population Medicine, University of Minnesota Twin Cities, St. Paul, MN, United States

**Keywords:** editorial, foot and mouth disease, vaccines, vaccination, epidemiology, vaccine efficacy assessment

Vaccination has played a major role in foot-and-mouth disease (FMD) control. There are different approaches to the design and implementation of vaccination campaigns, and epidemiological information is paramount in influencing the vaccine and vaccination strategy that best suit each geographic location. FMD-endemic regions typically organize vaccination campaigns as a routine preventive control policy or to mitigate the impact of the disease. The majority of currently used vaccines are formulated with chemically inactivated whole-viral particles and suitable adjuvants such as single and double oil emulsions. The most recent strains circulating in a particular region are typically selected as antigens based on the results of vaccine-matching data and *in vitro* experiments, however, predictions based on vaccine-matching approaches are usually uncertain without a live virus challenge in natural hosts combined with reliable field data. Vaccine selection and successful vaccination campaigns rely on a deep knowledge of the epidemiology of the region where these vaccines will be used, as well as access to the appropriate diagnostic tools to underpin these campaigns.

Inactivated vaccines are produced by growing large amounts of live virus, which requires facilities with high biosecurity levels and poses a risk of virus escape that may hinder vaccine production in FMD-free areas. In addition, inadequate inactivation of the antigen used to formulate vaccines could potentially cause outbreaks, so a residual risk may persist if the process does not follow adequate quality standards. New-generation vaccines that can be produced without culturing fully infectious virus could provide a solution to these risks. Ideally, these vaccines should protect the host against a vast number of FMD strains and provide at least the same level of protection compared to current, inactivated vaccines.

The main objective of this Research Topic was to gather studies focussed on aspects of FMD vaccine and vaccination to advance the science supporting the implementation of vaccination campaigns that assist the prevention and control of the disease.

This Research Topic hosted by scientists networking through the Global Foot and Mouth Disease Research Alliance (GFRA—https://www.ars.usda.gov/gfra/) includes 14 manuscripts that cover a variety of studies that investigate and discuss the diverse research gaps in FMD vaccine and vaccination; including the progress of FMD control programs in different parts of the world, control measures design and follow-up, risk-assessments for vaccine use, vaccine strain selection, immune responses to currently used vaccines in different species and tools for novel vaccine design, among other issues.

## Progress of FMD control programs

Three studies were aimed at evaluating the progress of FMD control programs in which vaccination strategies were used.

Cabezas et al. introduced a retrospective analysis that described the suspensions and recoveries of 45 FMD-free status in the World Organization for Animal Health (WOAH) Members States and evaluated the impact of several risk factors on the time to recover FMD-free status. Most of the FMD-free status suspensions (>50%) were in the Americas and Africa, and about 70% of these suspensions occurred in previously free without vaccination areas. The study noted that implementing a stamping-out or vaccination and removal policy reduced the time to recover FMD-free status, compared with a vaccination and retain policy due to additional requirements for post-outbreak surveillance. Nevertheless, this study confirms once again that vaccination plays a key role in the control of FMD emergencies.

The success of the Progressive Control Pathway (PCP) strategy in Kazakhstan achieving freedom from FMD by combining zoning, movement control, vaccination, and surveillance was described by Sultanov et al.. The study provides an overview of the key factors leading to the successful control of the disease in the country and offers a discussion that is of interest to other countries in the Central Asia region in which FMD is yet to be controlled.

A review of the progress of South America toward FMD eradication (Rivera et al.) was presented, accounting for more than 70 years of dealing with the disease and 35 years of well-organized public-private partnership that finally is leading the region to eradication by 2025. This productive regional governance was accompanied with high-quality and batch-controlled oil vaccines (most of them locally produced), improved surveillance of post-vaccination immunity that permitted to strengthen of control measures in immunologically resized subregions, and the reinforcement of the capacity of the veterinary service and control of animal movement in most of the countries within a particular region. Currently, many territories have taken the step toward withdrawing the vaccine and being recognized as FMD-free without vaccination by the WOAH. This success demonstrates the importance of regional cooperation to control FMD successfully.

## Advances in FMD epidemiology

Shurbe et al. provided evidence for using vaccinations in the field through observational or simulation epidemiology approaches. The authors collected and assessed data in Southern Ethiopia, confirming virus circulation, and analyzing risk factors and the socio-economic impact of the presence of FMDV in the region, which is a prerequisite for the design and application of operative control programs in the field. Also critical to accomplish FMDV epidemiological studies, a new approach for a well-known diagnostic tool was developed. A TaqMan-based real-time reverse transcriptase PCR is presented by Chestley et al. using bioinformatics to specifically identify the Southern African Territories (SAT) 1, 2, and 3 serotype strains, excluding other FMDV strains circulating in the region.

Two reports followed modeling approaches to study specific aspects of the vaccine-based controlled strategies. Yadav et al. investigated the economic and epidemiologic impacts of the vaccination-to-live strategy in FMD-free regions. Different scenarios of disease spread, and control were created using the US livestock population as a model. The authors report that production losses were superior when outbreaks began simultaneously in multiple sites, but smaller when compared to trade and consumer avoidance losses. The model predicted a high percentage of potentially persistently infected animals, arising from infected animals in the vaccinated population and discusses the deployment of appropriate post-outbreak management strategies.

An alternative modeling approach was used by Yang et al. to study the impact of different vaccination parameters in managing the disease and comparing the efficacy of the vaccines vs. the vaccine coverage in the field. The authors conclude that increasing vaccine efficacy has a deeper impact on vaccine-based strategies than increasing vaccine coverage.

## Vaccine efficacy

Vaccine antigen selection methods and vaccine efficacy in susceptible species were also examined.

Vaccine dose and vaccination schedules optimization in different target species were analyzed in a study performed in Mongolia (Ulziibat et al.). This field study compared the capacity of a two-dose or a single double-dose vaccination of inducing protective levels of neutralizing antibodies and concluded that a single double dose will provide similar results to the traditionally used scheme while being more cost-effective.

A study by Horsington et al. evaluated the protective ability of a bivalent vaccine of different South Asia lineage serotype A strains against the A/Asia/SEA-97 variants in pigs, instead of using the same strains as monovalent preparations. Improved protection with an increased number of virus strains has been shown before, explaining their success due to the availability of a higher number of conserved epitopes available to the immune system (1).

These challenge studies are paramount to provide information that can be used to feed models that can help select antigens without the need of challenging animals with live virus. Laboratory tools with the optimized capacity to score the adequacy of vaccine-induced immunity were also evaluated based on vaccine performance data from the field (Gubbins et al.). In this regard, Ludi et al. presented “PRAGMATIST,” a semi-quantitative FMD strain selection tool that uses information on vaccine efficacy trials, laboratory vaccine matching results and risk scores. The authors highlighted the variation in the vaccine antigens required for storage in FMD-free regions where vaccination is not applied.

## Tools for new generation vaccine development

Virus-antibody interactions are also studied to further optimize vaccines and improve quality control (Harmsen et al.), based on the detection of FMDV capsid integrity. Also in this collection, Summerfield et al. delved into the relationship between opsonizing and neutralizing monoclonal FMDV-specific antibodies and assigned a role for low avidity antibodies, as their interactions are enough to mediate Fcγ receptor-mediated functions that could play a role in the protective immunity against FMDV. Harmsen et al. provided knowledge of the particle specificity of VHHs that can be applied to the production of VLPs with improved immunogenicity.

VLPs and live-attenuated vaccines are new-generation vaccine candidates that can be grown in low biosafety environments. An article by Azzinaro et al. supports the view that manipulation of the Lpro coding region can provide a tool to develop FMDV live attenuated strains of FMDV while ensuring that sufficient replication is achieved to induce a protective and sustained immune response. Moreover, the incorporation of mutations that could stabilize attenuating mutations and prevent recombination with circulating viruses, is an important requirement for the success of such an approach.

## Conclusions

Altogether, the articles in this collection bring an overview of the current advances in FMD vaccine development, vaccine selection, vaccination, and epidemiology research to produce tools for FMD control and the pathway for eradication. There remains much to achieve, especially in understanding cross-protection, vaccine strain selection and how to perform accurate risk assessments. These gaps can be closed by promoting collaboration between groups working on FMD globally, supported by international initiatives such as the Global Foot and Mouth Disease Research Alliance.

This Research Topic includes articles that improve our capacity of using vaccination as a key tool to prevent and control FMD, contributing to the sustainability of the livestock industry worldwide.

## Author contributions

All authors listed have made a substantial, direct, and intellectual contribution to the work and approved it for publication.
